# Successfully treated with siltuximab and prednisone in a 7-year-old girl with *DOCK8*-deficiency presenting as recurrent wart-like lesions: a case report

**DOI:** 10.3389/fimmu.2024.1414573

**Published:** 2024-07-09

**Authors:** Zhe Sun, Ruoqu Wei, Chaolan Pan, Cheng Ni, Xue Zhang, Wenbin Guan, Ruhong Cheng, Yan Gu, Hong Yu, Kejun He, Zhen Zhang, Xia Yu, Zhirong Yao

**Affiliations:** ^1^ Dermatology Center, Xinhua Hospital, Shanghai Jiaotong University School of Medicine, Shanghai, China; ^2^ Department of Dermatology, Shanghai Jiaotong University School of Medicine, Shanghai, China; ^3^ Institute of Dermatology, Shanghai Jiaotong University School of Medicine, Shanghai, China; ^4^ Department of Allergy, Dermatology Center, Xinhua Hospital, Shanghai Jiaotong University School of Medicine, Shanghai, China; ^5^ Department of Pathology, Xinhua Hospital, Shanghai Jiaotong University School of Medicine, Shanghai, China; ^6^ Department of Pediatric Hematology and Oncology, Xinhua Hospital, Shanghai Jiaotong University School of Medicine, Shanghai, China

**Keywords:** DOCK8 deficiency, herpetic infections, IL-6, siltuximab, primary immunodeficiency

## Abstract

Dedicator of cytokinesis 8 (DOCK8) deficiency represents a primary immunodeficiency with a wide range of clinical symptoms, including recurrent infections, atopy, and increased malignancy risk. This study presents a case of a 6-year-old girl with *DOCK8* deficiency, characterized by severe, treatment-resistant herpetic infections who was successfully treated with siltuximab and glucocorticoids. The successful use of siltuximab in achieving remission highlights the pivotal role of interleukin-6 (IL-6) in *DOCK8* deficiency pathogenesis and suggests that IL-6 modulation can be critical in managing *DOCK8* deficiency-related viral infections, which may inform future therapeutic strategies for *DOCK8* deficiency and similar immunodeficiencies.

## 1 Introduction

Type 2 autosomal-recessive hyper-IgE syndrome (AR-HIES2) (OMIM 243700) is a combined immunodeficiency that exemplifies the broad clinical features of IEI (inborn errors of immunity) (1). Mutations in the gene encoding DOCK8 account for the majority of patients with AR-HIES2, referred to as *DOCK8* deficiency, characterized by eosinophilia, elevated immunoglobulin E (IgE) levels, recurrent infections, atopic dermatitis (AD), allergies, autoimmunity and malignancy (2, 3). The DOCK8 protein plays an important role in cytoskeletal organization, affecting dendritic cell migration, the proliferation and apoptosis of T cells and B cells, and natural killer cell toxicity (4, 5). It causes susceptibility to cutaneous viral infections, especially with herpes simplex virus (HSV), human papillomavirus (HPV), molluscum contagiosum virus (MCV), and varicella-zoster virus (VZV) (1). These infections may be severe, persistent, and refractory to treatment, representing a major cause of morbidity and at times mortality in *DOCK8* deficiency. Here, we describe a patient with *DOCK8* deficiency and severe, progressive herpetic infections refractory to therapy with valacyclovir. Our patient got surprising relief after combination treatment of siltuximab and glucocorticoid in addition to antiviral treatment, suggesting a pivotal role of IL-6 in pathogenesis.

## 2 Case presentation

**Figure 1:**
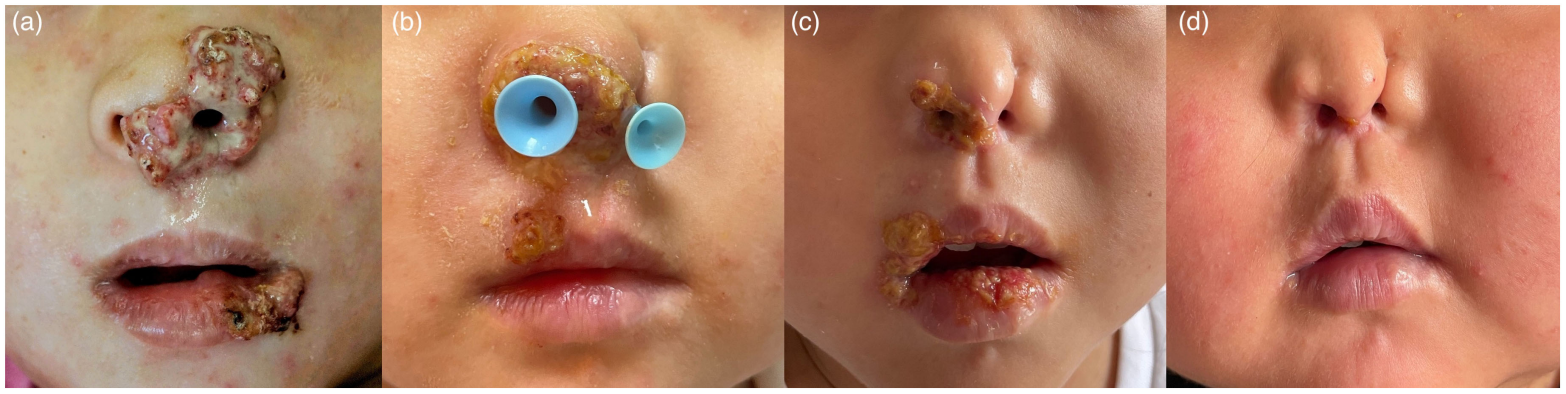
Representative pictures of the lesions in patient early in their infection. **(A)**, after IFN-a therapy **(B)**, after 4 doses of siltuximab **(C)**, and 6 months after treatment **(D)**, respective.

A 6-year-old Chinese girl, presenting with a two-year history of wart-like masses on her nose and lip, was referred to our dermatology clinic in May 2021. At age 4, a red papule appeared on her left side of nose, progressed in size and recurred shortly after they were excised three times with the pathological diagnosis of hemangioma in other hospitals. Gradually, asymptomatic granulomatous masses with purulent exudation in the right side of oral cavity developed in the recent 6 months (**Figure 1A**). The patient didn’t have any constitutional symptoms including fever, night sweats, fatigue and weight loss. She was born of a consanguineous marriage and exhibited a clinical history notable for recurrent respiratory tract infections, AD and foods allergies to milk, eggs, and seafood.

**Figure 2:**
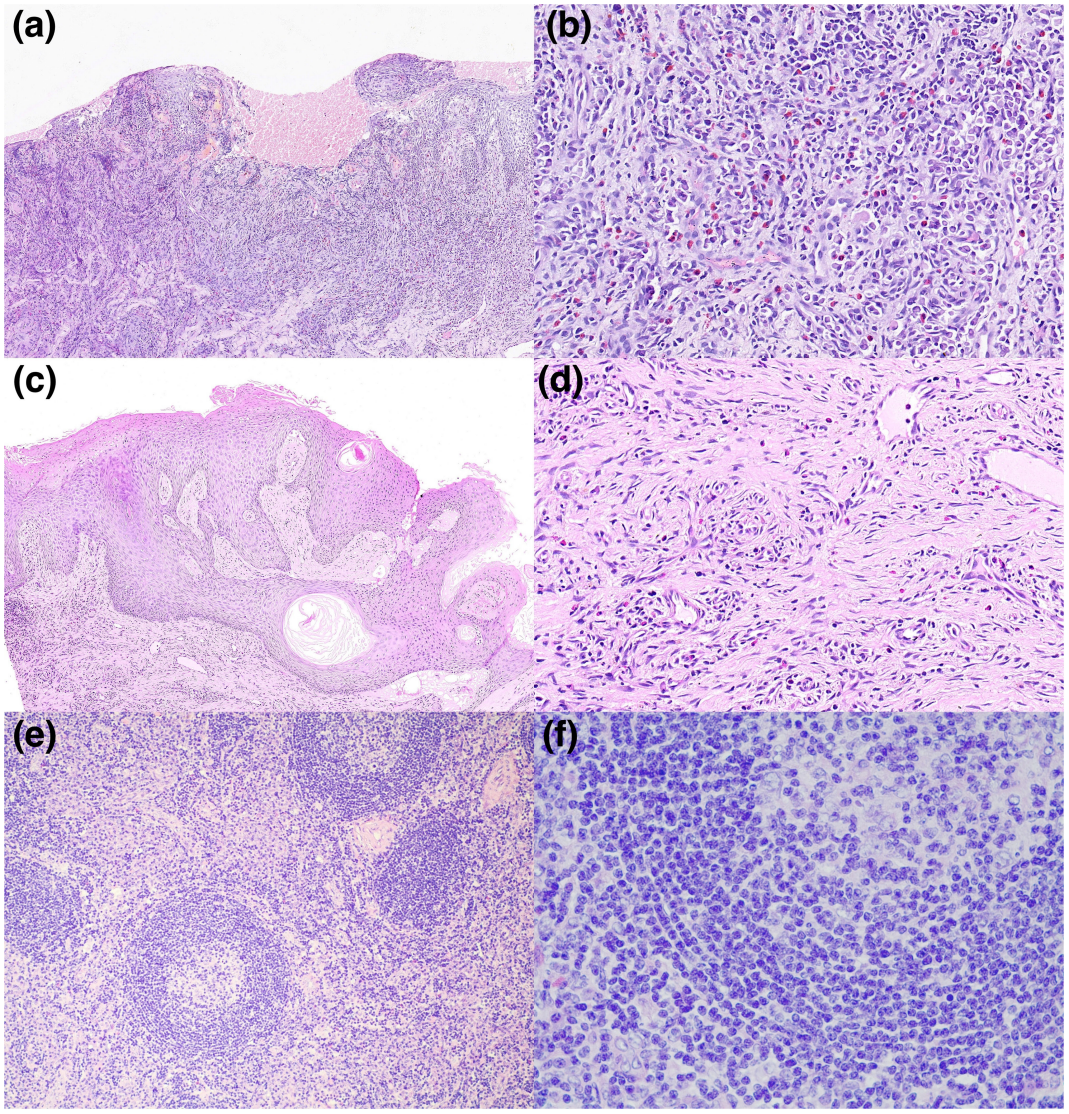
**(A–F)** Microscopies of skin biopsy specimens, hematoxylin and eosin (H&E). **(A, B)** Biopsy of nose displayed local ulceration of squamous epithelium with inflammatory exudation, granulation tissue hyperplasia and squamous pseudoepithelioma. A significant infiltration of neutrophils, eosinophils, and plasma cells is evident. Degenerative changes in the nuclei of the squamous epithelial cells, particularly at the site of the ulceration, aligns with the histopathological hallmarks consistent with HSV infection. **(C, D)** Biopsy of oral cavity showed superficial and deep perivascular inflammation with cavernous edema. **(E, F)** Biopsy of the enlarged right submandibular lymph node revealed follicular hyperplasia, partly thickening of follicular mantle zone, and proliferation of plasma cells in the lymphoid interlobular area, resembling Castleman’s disease. (**A**: H&E, x100; **B**: H&E, x400; **C**: H&E, x100; **D**: H&E, x400; **E**: H&E, x100; **F**: H&E, x400).

Physical examination revealed multiple enlarged lymph nodes in submental, submandibular and neck region. Laboratory results were presented in **Table 1**. Human immunodeficiency virus and HHV-8 serologies were negative. We took the nasal lesion for pathology and revealed an epithelioid hemangioma (**Figures 2A, B**). Biopsy from oral cavity showed superficial and deep perivascular inflammation with cavernous edema (**Figures 2C, D**). The pathology of enlarged right submandibular lymph node revealed reactive lymphoid hyperplasia with Castleman-like histology, including follicular hyperplasia, partly thickening of follicular mantle zone, and proliferation of plasma cells in the lymphoid interlobular area (**Figures 2E, F**). HSV-1 was confirmed by immunohistochemical analysis of the nasal lesion.

**Table 1:** Complete blood count and biochemical and immunological features of the patient.

	Normal range	Before treatment	After the fourth therapy	After the sixth therapy
Leukocyte(*10^9/L)	4-10	7.47	**11.66**	9.88
Eosinophils (*10^9/L)	0-0.68 (0.5-5%)	**1.12** **(15.0%)**	0.17 (1.5%)	0.17 (1.7%)
Platelet count(*10^9/L)	100-300	**473**	264	254
**Albumin (g/L)**	35-50	**20.1**	**34.2**	42.7
Immunoglobulins				
**IgG(g/L)**	7-16	**4.48**	**2.49**	**4.51**
**IgA(g/L)**	0.7-4.0	0.88	**0.65**	0.84
**IgM(g/L)**	0.4-2.3	0.41	0.73	0.82
**IgE(IU/mL)**	0-100	**16700**	**2430**	**2100**
Lymphocyte subsets				
**CD3(/uL)**	60.8-75.4% (1141-1880)	**43.11%** **(513.39)**	**18.97%** **(265.63)**	**22.39%** **(151.55)**
**CD4(/uL)**	29.4-45.8% (478-1072)	32.88% **(391.61)**	**13.43%** **(188.10)**	**18.59%** **(125.82)**
**CD8(/uL)**	18.2-32.8% (393-742)	**7.37%** **(87.75)**	**3.72%** **(52.11)**	**3.17%** **(21.49)**
**CD19(/uL)**	6.8-15.8% (117-332)	**51.23%** **(25.67)**	**74.95%** **(1049.80)**	**68.96%** **(466.80)**
**CD20(/uL)**	60.8-75.4% (1141-1880)	**50.74%** **(597.64)**	73.61% **(1031.03)**	68.16% **(461.38)**
**CD16 + 56(/uL)**	9.5-23.5% (175-567)	**2.16%** **(25.67)**	**2.54%** **(35.59)**	**7.56%** **(51.18)**
**CD4/CD8(%)**	0.98-1.94	**4.46**	**3.61**	**5.86**
**IL-6(pg/ml)**	<5.9	**11.20**	**6.15**	2.88

Bold values indicate abnormal indicators which are helpful to read.

**Figure 3:**
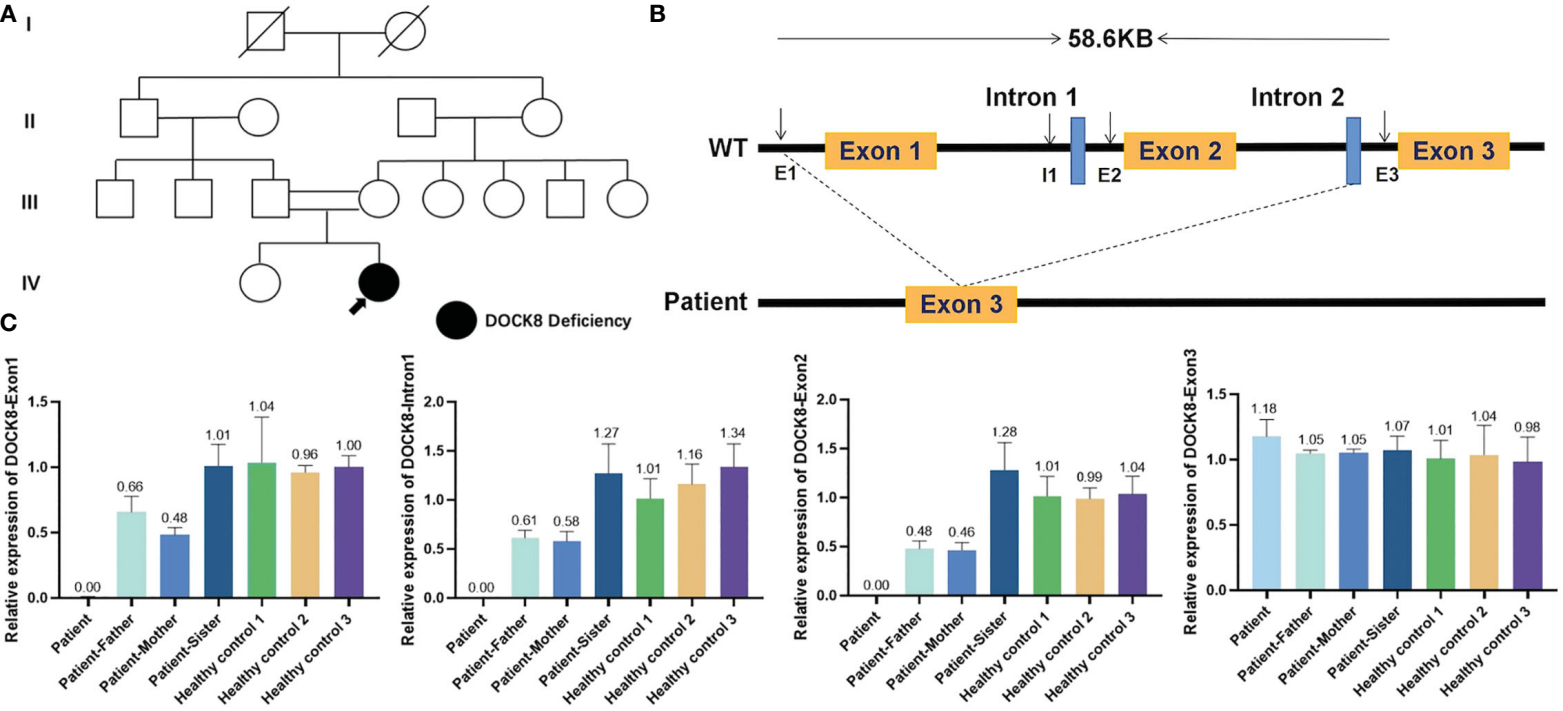
Deletion in dedicator of cytokinesis 8 (*DOCK8*). **(A)** Pedigree of a Chinese family with AR-HIES2. Filled symbol represents the patient. Open symbols represent unaffected individuals. The double lines represent a consanguineous union. Her parents and sister were all healthy. **(B)** Schematic representation of the wildtype *DOCK8* gene, which comprises 48 exons. The patient has a homozygous mutation in the *DOCK8* gene, which was a 58.6Kb genomic deletion in exons 1–2 inherited from her parents. **(C)** To verify whether the proband, her parents and sister had the deletion, we performed qRT-PCR for exon1, Intron1, exon2, Intron2 and exon3 (which was contained in this ~58.6Kb-size deletion) of *DOCK8*. RNA was isolated from the peripheral blood of the patient, her parents, her sister and three healthy controls by the RNAzol method. CT values were compared with that of ALB by the relative quantification system. The proband and her parents showed the deletion (chr9:214506-273133) in exon 1 and exon 2 of *DOCK8*.

Approval was obtained from the ethics committee of Xinhua Hospital, Shanghai Jiaotong University School of Medicine, and all procedures were performed according to the principles of the Declaration of Helsinki. To identify whether the patient had primary immunodeficiency, whole-exome sequencing (WES) was performed after obtaining written informed consent. WES revealed a homozygous mutation in the *DOCK8* gene, which was a ~58.6Kb genomic deletion in exons 1–2 inherited from her parents. We performed qRT-PCR for exon1, Intron1, exon2, Intron2 and exon3 (which was contained in this ~58.6Kb-size deletion) of *DOCK8*, and the proband and her parents showed the deletion (chr9:214506-273133) in exon 1 and exon 2 of *DOCK8* (**Figure 3**). Combined with the clinical manifestations, laboratory results and WES findings, AR-HIES2 was established.

The patient had experienced various treatments. Oral valaciclovir and intravenous immunoglobulin (1g/kg every month) were prescribed for 2 months, and the exudation of nose and oral cavity reduced while there was no visible shrinkage of granulomatous masses. As the granulomatous mass on the nose corroded the nasal cavity, we conducted surgical resection again but relapsed one month later (**Figure 1B**). Pegylated interferon-alpha 2a (IFNα-2a) through topical and subcutaneous injection were applied but had little effect. In light of persistent symptoms, the pathology of Castleman-like lymph node and high IL-6 levels, the patient was administered an IL-6 inhibitor, siltuximab (200mg every 3 weeks) and prednisone(1.5mg/kg/day) in addition to antiviral treatment. All other treatments mentioned above were discontinued due to their limited effects. Remarkably, this regimen induced a significant reduction in symptoms and lymph node size during the fourth therapy (**Figure 1C**). As we found CD3, CD4 and CD8 counts were low in our patient (**Table 1**), we administered trimethoprim-sulfamethoxazole for pneumocystis jirovecii prophylaxis, at a dosage of 0.24g twice daily, three times a week. The family refused further immunoglobulin replacement therapy due to the high cost and limited immediate effect. Prednisone was reduced by half and complete remission was achieved at the sixth therapy (**Figure 1D**). Except for IgE, eosinophils and IL-6 were within the normal range. We gave her thymalfasin and valaciclovir for immune enhancement. Prednisone was gradually tapered to a final dose of 4 mg every other day, starting with 8 mg daily for the first two months and 4 mg daily for the next two months, resulting in a sustained response throughout the six-month follow-up period. Although HSCT was suggested as a definitive treatment, it was not pursued due to economic factors.

## 3 Discussion

*DOCK8* deficiency is a rare IEIs seen predominantly in consanguineous populations, now recognized as a distinct form of combined immunodeficiency (2). It impairs cytotoxic CD8+ T-cell memory, NK T-cell populations and functionality, B-cell activation, antigen-specific response formation, and signaling via *DOCK8* and MyD88-dependent toll-like receptors (5, 6). The *DOCK8* gene has over 30 mutations identified across populations since 2009, with large deletions being most common, but also includes point mutations, splice site mutations, and small insertions/deletions, leading to little or no DOCK8 protein expression. There’s no significant difference in the phenotypic features of AR-HIES2 patients with various *DOCK8* mutation genotypes (7). Individuals with *DOCK8*-deficiency have a high incidence of allergic diseases and eczema, hyper IgE, eosinophilia, and a profound type-2 CD4+ T helper cell (Th2) bias (8), leading to IL-6 overexpression (9). An increased susceptibility to mucocutaneous viral infections, particularly caused by HSV, HPV, MCV, and VZV, is a hallmark of *DOCK8* deficiency, differentiating it from other hyper-IgE conditions (1). These DNA viruses establish latent infections after initial infections in the general population during childhood (2).

In the context of *DOCK8* deficiency, HSV infections are notably severe, often becoming chronic and leading to resistance against systemic therapies like acyclovir or valacyclovir (10). HSCT is advocated for all affected individuals, ideally at an early stage of the disease (6). But this procedure is associated with a high risk of morbidity and mortality particularly with uncontrolled viral infection. Interferon therapy has been shown to be effective by inhibiting viral replication and activating effector lymphocytes, including NK and cytotoxic T cells (11, 12). However, our patient was unresponsive to antiviral and interferon therapies. Elevated IL-6 levels and a Castleman-like lymph node pathology suggested the use of siltuximab, which is anti-human IL-6 monoclonal antibody approved for idiopathic multicentric Castleman’s disease. The successful use of siltuximab in achieving remission demonstrates the immunomodulatory role of IL-6 in antiviral defense against HSV in the context of *DOCK8* deficiency.

IL-6 plays diverse roles in plasma cell maturation, acute inflammatory mediator production, and vascular endothelial growth factor (VEGF) secretion (13, 14). HSV subverts the host’s immune system by encoding a homolog of human IL-6, referred to as viral IL-6 (vIL-6), in monocyte-derived mature dendritic cells (DCs) (15). High serum IL-6 levels enhance angiogenesis and increase vascular permeability by upregulating VEGF expression (16) and induces cytokine storms (13, 17). It might also contribute to B-cell proliferation and angiogenesis within lymph nodes via IL-6R-independent engagement of gp130. In rodent experiments, IL-6 transgenic mice displayed multiple lymph node swelling and follicular hyperplasia (18). In our patient, VEGF was strongly expressed in the plasma cells around the hyalinized vessels in the interfollicular region, and the pathology described as hemangioma verified our hypothesis that virally expressed VEGF may be responsible for proliferative granulomatous masses. Anti-IL-6 therapy may act by reducing cytokine storms driven by high serum IL-6 levels and inhibiting HSV replication. Clinical response to siltuximab observed in this individual also supports this contention. However, the multifaceted nature of IL-6’s actions highlights the need for a nuanced understanding, considering various regulatory factors. Further studies are needed to fully understand the specific mechanisms of IL-6 for advancing our knowledge and potential therapeutic interventions.

In summary, this report details a pediatric case of *DOCK8* deficiency accompanied by severe, progressive herpetic infections non-responsive to standard antiviral and interferon therapies, yet responsive to siltuximab and glucocorticoid administration. Our findings suggest that IL-6 serves as a crucial modulator of immune response to HSV with implications for its regulatory capacity in inflammation, cellular, and humoral immunity, warranting further exploration into its specific mechanistic pathways. Anti-IL-6 therapy may be beneficial for *DOCK8*-deficient subjects who prove unresponsive to conventional antiviral chemotherapies, indicating a prospective avenue for clinical management of this immunodeficiency.

## 4 Conclusion

Our case describes a 6-year-old patient with *DOCK8* deficiency and severe, progressive herpetic infection. Given the distinct clinical phenotype of *DOCK8* deficiency, characterized by eczema, recurrent bacterial skin and lung infections, chronic viral skin infections, and severe allergies in combination with a cellular immunodeficiency and increased risk for malignancy, it would seem straightforward to diagnose affected patients early. The severe cutaneous phenotype in our patient responded to a combination of siltuximab and prednisone in addition to antiviral treatment. Our research findings indicate that IL-6 plays a crucial regulatory role in the immune response against HSV, demonstrating its role in inflammation regulation, cellular immunity, and humoral immunity. Anti-IL-6 therapy may be beneficial for DOCK8 deficient subjects who have been shown to be unresponsive to traditional antiviral chemotherapy. Our findings suggests that IL-6 emerges as a pivotal regulator in the immune response against HSV, demonstrating its roles in inflammation modulation, cellular immunity, and humoral immunity. Anti-IL-6 therapy may be beneficial in those *DOCK8-*deficient subjects who prove unresponsive to conventional antiviral chemotherapies.

## Data availability statement

The data presented in the study are deposited in the GeneBank repository, accession number BioSample: SAMN41533898 and SRA: SRR29181171.

## Ethics statement

The studies involving humans were approved by Ethics Committee of Xinhua Hospital, Shanghai Jiaotong University School of Medicine. The studies were conducted in accordance with the local legislation and institutional requirements. Written informed consent for participation in this study was provided by the participants’ legal guardians/next of kin. Written informed consent was obtained from the individual(s), and minor(s)’ legal guardian/next of kin, for the publication of any potentially identifiable images or data included in this article. Written informed consent was obtained from the participant/patient(s) for the publication of this case report.

## Author contributions

ZS: Conceptualization, Data curation, Formal analysis, Funding acquisition, Investigation, Methodology, Project administration, Resources, Software, Supervision, Validation, Visualization, Writing – original draft, Writing – review & editing. RW: Conceptualization, Data curation, Formal analysis, Funding acquisition, Investigation, Methodology, Project administration, Resources, Software, Supervision, Validation, Visualization, Writing – original draft, Writing – review & editing. CP: Conceptualization, Data curation, Formal analysis, Funding acquisition, Investigation, Methodology, Project administration, Resources, Software, Supervision, Validation, Visualization, Writing – original draft, Writing – review & editing. CN: Writing – review & editing, Conceptualization, Data curation, Investigation, Project administration, Validation. XZ: Writing – review & editing, Data curation, Funding acquisition, Investigation, Methodology, Validation. WG: Writing – review & editing, Data curation, Formal analysis, Investigation, Methodology, Software. RC: Writing – review & editing, Conceptualization, Data curation, Investigation, Methodology, Software, Supervision. YG: Investigation, Methodology, Software, Writing – review & editing, Formal analysis. HY: Formal analysis, Investigation, Methodology, Software, Writing – review & editing. KH: Investigation, Methodology, Project administration, Supervision, Writing – review & editing. ZZ: Conceptualization, Data curation, Formal analysis, Funding acquisition, Investigation, Methodology, Project administration, Resources, Software, Supervision, Validation, Visualization, Writing – review & editing. XY: Conceptualization, Data curation, Formal analysis, Funding acquisition, Investigation, Methodology, Project administration, Resources, Software, Supervision, Validation, Visualization, Writing – review & editing. ZY: Conceptualization, Data curation, Formal analysis, Funding acquisition, Investigation, Methodology, Project administration, Resources, Software, Supervision, Validation, Visualization, Writing – review & editing.
